# Answers to Correspondents

**Published:** 1908-08-29

**Authors:** 


### Editor's Letter-Box.

ANSWERS TO CORRESPONDENTS.

Lebanon.—The presence of your name in the official "Medical Register" for the current year is legal evidence of your status as a registered medical practitioner, and is accepted as such in all Courts of Law. The "Medical Register" is published early in each year by the General Medical Council, and must not be confused with the medical Directories, which are private undertakings, issued solely for the convenience of the profession and the public. The General Medical Council also publishes an annual "Dentist's Register." The official "Registers" are arranged in alphabetical order of names, irrespective of locality, and contain no personal information beyond the practitioners' full name and address, his registered qualifications, the dates of qualifications, and the place and date of registration. The fee for registering an additional qualification is £1 for each new diploma or degree; but there is no obligation on your part to register your recent degree, since you registered your original qualifications three years ago; nevertheless, it is well to do so.

Leicester.—Write at once to the Dean of the Medical School, and in the meantime obtain a copy of the birth certificate. The next issue of The Hospital, published on Saturday, September 15, will be the annual Educational Number, and will contain most of the other information that you desire.

